# PReS-FINAL-2305: Erythema multiforme in a child with lupus - what's in a name?

**DOI:** 10.1186/1546-0096-11-S2-P295

**Published:** 2013-12-05

**Authors:** R Khubchandani, AD Dhanrajani, DA Parikh

**Affiliations:** 1 Pediatrics, Jaslok Hospital And Research Center, Mumbai, India; 2 Dermatology, Bombay Hospital, Mumbai, India

## Introduction

The occurrence of Erythema Multiforme (EM) like lesions in lupus has been reported in 98 patients to date but only in 9 children below 18 years.

## Objectives

To sensitize pediatric rheumatologists about this facet of lupus through a picture essay of our case.

## Methods

Case report with a picture essay.

## Results

A 16 year old girl diagnosed as lupus 2 months ago with fever, butterfly facial rash, mucositis and arthritis as the only clinical features and a strongly positive ANA, presented to us with a sub-acute (over 7 - 10 days), progressive photosensitive mucocutaneous eruption that was erythematous maculopapular, bullous with annular plaques distributed over the face, neck arms and legs, relatively sparing the scalp, palms and soles. Oral ulcers were present on the inside of the lower lip. She had high fever and significant constitutional features. There was no history of ingestion of any incriminating drugs. She was on low dose oral steroids and hydroxychloroquin at the time of presentation.At this stage her investigations revealed Hemoglobin of 8.4 gm/dl, Total WBC counts 12,680/cu.mm, platelet count 1,90000. CRP was negative.C3 levels were 28 mg/dl, but rheumatoid factor (RF), Anti - Ro and Anti - La antibodies were negative. She was started on pulse methyl prednisolone at a dose of 30 mg per kilogram body weight for 3 days which led to a rapid reduction of rash and constitutional symptoms. We discharged her on oral prednisolone (2 mg/kg) and azathioprine (2 mg/kg). On follow up at 4 weeks just days before this submission her skin lesions had almost cleared. She continues follow up and her updated status will be presented.

## Conclusion

The presence of EM like lesions in Lupus when associated with a certain immunological profile (positive RF, anti Ro and La) was first described in 1963 and later christened as Rowells syndrome. Confusion surrounds the existence of this entity with two articles as recently as late 2012 disputing its existence and disagreeing regarding terminology and classification.

Pediatric cases are rare and irrespective of the debates on nomenclature and classification the clinician should learn to recognize the occurrence of this severe morphological manifestation of cutaneous lupus without splitting hair on nosology.

Treatment is with steroids and azathioprine in addition to antimalarials. Dapsone and Cyclosporin have also been used.

The limitation of our report is that we did not exclude the possibility of Mycoplasma and Herpes Simplex viruses in the work up and it remains speculative whether hydroxychloroquin could be the incriminating drug.

## Disclosure of interest

None declared.

